# Evaluation of Biological Pretreatment of Wormwood Rod Reies with White Rot Fungi for Preparation of Porous Carbon

**DOI:** 10.3390/jof9010043

**Published:** 2022-12-28

**Authors:** Wen Kong, Shuhui Wang, Xinyu Zhang, Xiao Fu, Wanju Zhang

**Affiliations:** Hubei Key Lab for Processing and Application of Catalytic Materials, LiShizhen College of Traditional Chinese Medicine, Huanggang Normal University, Huanggang 438000, China

**Keywords:** wormwood rod residues, white rot fungi, porous carbon

## Abstract

In this work, the wormwood rod residues are pretreated with white rot fungi as the precursor to preparing porous carbon following a simple carbonization and activation process (denoted herein as FWRA sample). The FWRA sample possesses abundant hierarchical pores structure with high specific surface area (1165.7 m^2^ g^−1^) and large pore volume (1.02 cm^3^ g^−1^). As an electrode for supercapacitors, the FWRA sample offers a high specific capacitance of 443.2 F g^−1^ at 0.5 A g^−1^ and superb rate ability holding a specific capacitance of 270 F g^−1^ at 100 A g^−1^ in 6 M KOH electrolyte. The corresponding symmetrical capacitor has a superb cyclic stability with a low specific capacitance decay rate of 0.4% after 20,000 cycles at 5 A g^−1^ in 1 M Na_2_SO_4_ electrolyte. Moreover, measurements revealed that when used as adsorbent, the FWRA sample is ideal for removing methyl orange (MO) from water, exhibiting a superior adsorption ability of 260.8 mg g^−1^. Therefore, this study is expected to provide a simple and environmentally friendly technique for the generation of value-added and functional porous carbon materials from Chinese medicinal herbal residues, thus offering promising candidates for broad application areas.

## 1. Introduction

Porous carbon materials with large specific surface areas and pore volumes, unique morphologies, controllable porous structures, excellent thermal, chemical, and mechanical stabilities have demonstrated exceptional performance in a variety of energy- and environment-related applications [1]. In the past decade, various carbon materials have been investigated and applied in many fields such as energy conversion and storage [2,3], electrode materials for supercapacitors [4,5], water pollutants adsorption [6,7,8], and so forth.

As biological material from living organisms, biomass is a source of renewable energy and can be obtained from agriculture waste, forestry waste, livestock and so on [9,10]. The biomass is composed primarily of cellulose, hemicelluloses and lignin and smaller amounts of pectin, protein, extractives and ash. Porous carbons are generally produced from coal or petroleum pitch. Due to concerns such as cost, availability and sustainability, researchers have been looking for alternatives [11]. Among the numerous carbon sources, biomass is considered highly suitable because it is renewable, broadly available, low cost and environmentally benign [9,12]. Lots of natural biomass materials such as cotton [13], barley [14], orange peels [15], potato peels [16], willow leaves [17] and so on, have been used to prepare porous carbons. In general, the most convenient method to develop biomass-derived porous carbons is by activation [1], but the involvement of harmful activating agents (e.g., strong acids or strong bases) hinders the wide use of this method. Hence, the development of simple, cost effective and pollution-free processes to obtain biomass-based carbon materials with a high specific surface area is still highly meaningful [18,19]. The structures of biomasses are complex. They are nanocomposites containing networks of cellulose and hemicellulose fibrils and complex “matrixing” polymers of lignin. Such structural features result in rigid structures and discontinuous channels that restrict the formation of pores and channels during carbonization and activation [20,21]. Inspired by the degradation process of biomass by fungi in nature, it is considered that hierarchical porous architectures may be created by fungi. Among natural biomass degrading fungi, the white rot fungi are mainly responsible for the lignin decomposition [22,23,24]. In the past, it was reported that the pretreatment with white rot fungi could improve the technological process of energy production from lignocellulosic biomass by reducing lignocellulose recalcitrance to enzymatic hydrolysis [25,26,27]. In addition, Cao and co-workers [28] synthesized ultrahigh-surface-area porous carbon by edible fungus slag, and Zhang et al. [7] fabricated hierarchical porous carbon from raw plant materials that had undergone fungal decomposition. *Physisporinus vitreus*, a white-rot basidiomycete, degrades lignin selectively and was tested for several biotechnological applications [27,29,30]. In the degradation process, the hyphae of white rot fungi infiltrate and biodegrade the internal structure of biomass substrate to construct porous structure, which is beneficial to the subsequent step of carbonization and chemical activation in terms of efficacy [7,21,28]. 

Chinese medicinal herbal residues (CMHR) is a solid plant biomass produced after the extraction of pharmaceutical ingredients from herb materials. At present, approximately 12 million tons of CMHR is produced annually from Chinese medicine enterprises. Nowadays, the treatment of CMHR mainly includes landfill, incineration, fixed-point open stacking and so on. These methods not only cost a lot of money, but also cause a waste of resources and serious environmental pollution [31,32]. It is essential to convert such biomass wastes to value-added products for a sustainable future. Nonetheless, to obtain useful carbon materials from CMHR with excellent properties, the selection of raw material as well as using synthesis method is critical. Wormwood (also named *Artemisia argyi*) is known as the “king of herbs” and is reputed for its strong vitality and wide applicability. It is one of the most important traditional Chinese medicines and has been used for over 2000 years. In general, the medicament portion of wormwood is its leaf rather than its rod. Therefore, the former has been widely studied while the latter has not. Wormwood rod is composed of cellulose, hemicellulose and lignin and contains elements such as carbon, oxygen, nitrogen and sulfur [33,34]. Hence, we envision that wormwood rod can be utilized for the generation of porous carbon materials. In this study, a comprehensive treatment method firstly pretreated by *P. vitreus* and then proceed with the carbonization and activation, was applied to generate wormwood rod residue based porous carbon materials in the lab. The electrochemical and adsorption performance of the synthesized carbon materials were compared with those of the carbon materials prepared without fungi pretreatment. The outcome not only demonstrates a simple and environmentally friendly technique, but also illustrates that fungi pretreatment is a promising strategy for the generation of value-added carbon materials from CMHR.

## 2. Materials and Methods

### 2.1. Materials and Chemicals

The wormwood rod residues were obtained from Qichun County, Hubei Province, China. White rot fungus *Physisporinus vitreus* was kindly provided by the Institute of Microbiotechnology of Environmental and Resources, Huazhong University of Science and Technology [27]. Sodium chloride (NaCl), hydrochloric acid (HCl), acetic acid (HAc), sodium acetate (NaAc), sodium sulfate (Na_2_SO_4_), potassium hydroxide (KOH), ethyl alcohol and methyl orange (MO) were purchased from Sinopharm Chemical Reagent Co., Ltd. (Shanghai, China) Polytetrafluoroethylene (PTFE) was bought from DAIKIN (Changshu, China), carbon black was obtained from Ketjenblack International Corporation (Tokyo, Japan), and nickel foam was procured from Changde (Beijing, China). All chemical reagents were of analytical grade and used as received without further purification. The distilled or deionized water was prepared in the laboratory.

### 2.2. Synthesis of Porous Carbon

#### 2.2.1. Synthesis of Wormwood Rod Residues Carbon (WR)

The wormwood rod residues were cut to 0.5–1.0 cm, and dried at 100 °C for 60 h to a constant weight for the removal of moisture and other volatile impurities. The dried wormwood rod residues were crushed with a small Chinese medicine grinder and screened over 80 mesh. The obtained powder was put into a porcelain boat and carried out in a tubular furnace and heated at 700 °C for 2 h under N_2_ atmosphere with a heating rate of 5 °C min^−1^. After natural cooling to room temperature, the as-resulted product was washed with 1 M HCl solution, then washed with distilled water for several times until neutral pH, and then dried at 120 °C overnight in a vacuum oven to obtain the WR.

#### 2.2.2. Synthesis of Carbon from White-Rot Fungi Treated Wormwood Rod Residues (FWR)

*P. vitreus* was cultured on potato extract agar slants for 7 days at 28 °C. Five disks (1 cm in diameter) of inocula were grown on potato extract broth for 5 days (150 revolutions per minute, 28 °C). The pretreatment of wormwood rod powder with *P. vitreus* was carried out in 250-mL Erlenmeyer flasks with 5 g of ground wormwood rod residues and 15 mL of distilled water. The flasks were sterilized in the autoclave for 30 min at 121 °C and aseptically inoculated with 5-mL fungal inocula. Cultures were maintained statically at 28 °C for 7 days, and then soaked by HAc-NaAc buffer solution, dried and calcined in N_2_ atmosphere at 700 °C for 2 h. According to the above procedures, FWR was obtained. 

#### 2.2.3. Synthesis of Carbon from White-Rot Fungi Treated Wormwood Rod Residues with NaCl Activated (FWRA)

The FWR was thoroughly mixed with NaCl (biochar: NaCl mass ratio = 1:2) and further pyrolyzed in a tubular furnace at 700 °C for 2 h in flowing N_2_ with a heating rate of 5 °C min^−1^. According to the above processing method, FWRA was obtained.

**Scheme 1:**
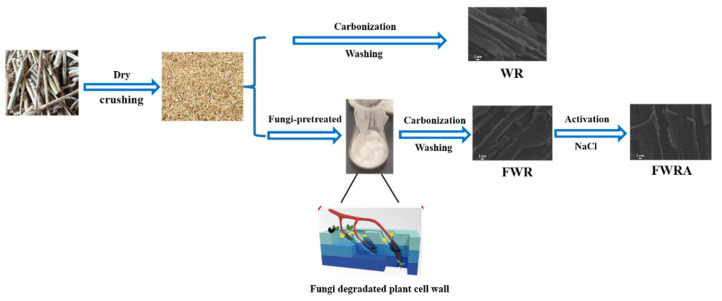
The scheme of preparation of porous carbons derived from wormwood rod residues.

The schematic of the preparation of porous carbons derived from wormwood rod residues is depicted in Scheme 1.

### 2.3. Characterization

The morphologies of samples were measured using scanning electron microscopy (SEM, Carl Zeiss Sigma 300, Roedermark, Germany). The crystal structures and defects of samples were examined via X-ray diffraction (XRD, Shimadzu XRD-6100, Kyoto, Japan) and Raman spectroscopy (Raman, InVia, Wotton-under-Edge, UK). The chemical compositions were confirmed using X-ray photoelectron spectroscopy (XPS, Thermo ESCALAB 250XI, Waltham, MA, USA). Surface area measurements were conducted on a surface area analysis and porosimetry analyzer (Micromeritics ASAP 2020, Norcross, GA, USA). All samples were degassed at 300 °C for 12 h in N_2_. With a backfill of N_2_, adsorption-desorption isotherms were generated at liquid nitrogen (77 K) bath. The surface area was calculated from the N_2_ adsorption isotherms by Brunauer-Emmett-Teller (BET) method. The pore size distributions (PSD) were obtained by Density Functional Theory (DFT) fitting of N_2_ adsorption data. 

### 2.4. Electrochemical Tests

The preparation of working electrodes was as follows: a mixture of 70 wt% carbon sample, 25 wt% carbon black and 5 wt% polytetrafluoroethylene (PTFE) was dispersed in alcohol and coated on nickel foam. The as-prepared electrodes were individually dried in an oven at 100 °C for 12 h. The active areas of the electrodes were about 1 cm^2^ and the quality of loading on the nickel foam was controlled within 0.7–1.1 mg cm^−2^. In the three-electrode system, 6 M KOH was used as the electrolyte solution, the above nickel foams individually loaded with one of the three samples were used as the working electrodes, Hg/HgO as the reference electrode, and platinum foil as the counter electrode. The capacitance performance was measured by cyclic voltammetry (CV), galvanostatic charge-discharge (GCD) and electrochemical impedance spectroscopy (EIS) on a CHI660E electrochemical workstation (Shanghai Chenhua Company, Shanghai, China). The specific capacitances (*C_s_*, F g^−1^) were determined from the galvanostatic charge-discharge curves according to the following Equation (1):

(1) $$C_{s}=\frac{I\Delta t}{m\Delta V}$$

In the two-electrode system, two identical working electrodes were assembled and 1 M Na_2_SO_4_ solution was used as the electrolyte. The cyclic stability of the electrodes of three samples was measured by using GCD curves acquired on a LANHE-CT2001A test workstation. The specific capacitance (*C_g_*, F g^−1^) of each electrode was calculated based on Equation (2):

(2) $$C_{g}=\frac{4I\Delta t}{m\Delta V}$$

The values of energy density (*E*, Wh kg^−1^) and power density (*P*, W kg^−1^) of the symmetric supercapacitor were calculated using Equations (3) and (4), respectively:

(3) $$E=\frac{C\Delta V^{2}}{7.2}$$

(4) $$P=\frac{3600E}{\Delta t}$$

where Δ*V* (V) is the cell potential change excluding voltage drop (IR drop), *m* (g) is the mass of active material on single electrode, *I* (A) is the discharge current, Δ*t* (s) is the discharge time.

### 2.5. Adsorption Tests

A large number of dye-containing waste waters were discharged from industrial processes without effective treatment. Methyl orange (a kind of azo dye) was selected in the present study to to determine the adsorption capacities of WR, FWR and FWRA in aqueous solution. A designated amount (i.e.,12.5 mg) of adsorbent samples was added to 50 mL of MO solution (50 mg/L) and stirred at 25 °C water bath. During the period of 0–180 min, the mixture was sampled at designated intervals, and the supernatant after centrifugation was analyzed at room temperature by Ultraviolet-visible spectroscopy at 463 nm to determine the concentration of MO in the aqueous phase. The amount of dye adsorbed on the adsorbent (*q*, mg/g) was calculated using Equation (5):

(5) $$q=\frac{(C_{0}-C_{e})V}{1000m}$$

where *C*_0_ and *C_e_* (mg/L) is initial MO concentration and equilibrium MO concentration, respectively, *V* (mL) is MO solution volume, *m* (mg) is the mass of added adsorbent. Every experiment was repeated at least three times and the average value was taken. 

## 3. Results

### 3.1. Morphology Characterization

**Figure 1:**
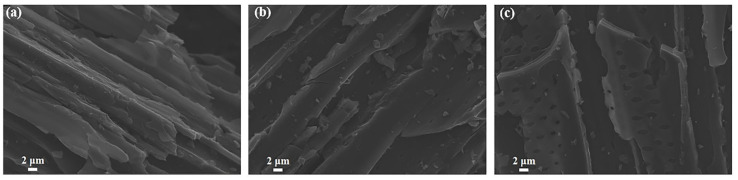
SEM images of (**a**) WR; (**b**) FWR; (**c**) FWRA.

To better explore the relationship between the biodegradation behavior of *P. vitreus* and the nature of porous carbon generated from the fungi treated wormwood rod, the morphologies of WR, FWR and FWRA were examined by scanning electron microscopy (SEM). Figure 1a showed that the non-inoculated WR sample is closely packed tubular channels and the surface is smooth with no visible pores or cavities. As for the FWR sample pretreated by white-rot fungi, it exhibits fragments broken off from the bulk, which are ten micrometers in size and porous in structure (Figure 1b). With the additional treatment of NaCl activation, the FWRA obtained from the FWR sample displays a 3D porous network and macroscopically open honeycomb structure (Figure 1c). 

### 3.2. Structure Characterization

**Figure 2:**
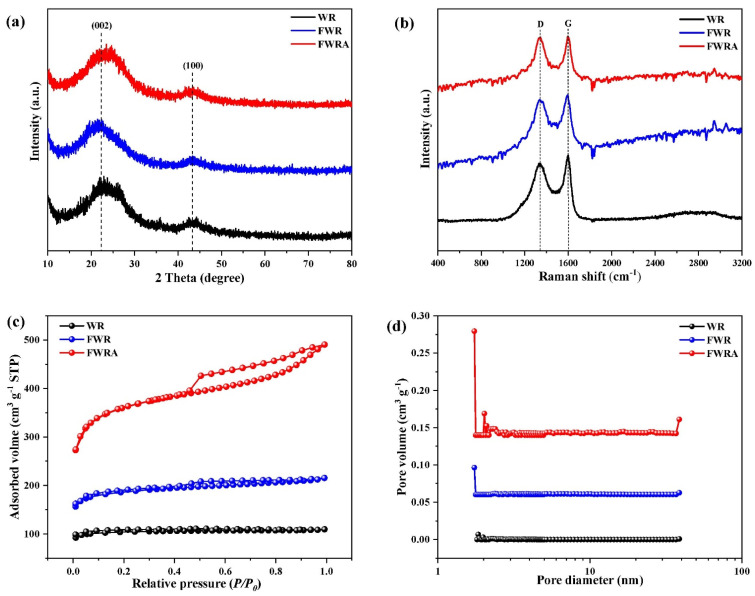
(**a**) XRD patterns; (**b**) Raman spectra; (**c**) N_2_ adsorption-desorption isotherm; (**d**) pore size distribution.

X-ray diffraction and Raman spectroscopy were used to analyze phase structure, confirming that WR, FWR and FWRA are mainly carbon materials. In Figure 2a, the diffraction peak at 2θ = 23° is attributed to the (002) planes of graphitic stacking and the peak at 2θ = 43° ascribed to (100) crystal plane, revealing disordered characteristics of the three materials [35]. Furthermore, in the Raman spectra of WR, FWR and FWRA (Figure 2b), there are two obvious feature peaks at around 1340 cm^−1^ (D-band) and 1580 cm^−1^ (G-band), which are caused by structural defects and graphitic sp^2^ carbon, respectively, indicating the existence of graphite structures. The I_G_/I_D_ values for WR, FWR and FWRA are 1.13, 1.02 and 1.01, respectively, suggesting a decreasing order of WR > FWR > FWRA in terms of graphitization level.

Figure 2c reveals the N_2_ adsorption-desorption isotherms of WR, FWR and FWRA. WR displays a typical type-I isotherm, which is characteristic of micropores. As for FWR and FWRA, they exhibit type-IV behavior with a distinct hysteresis loop at a high P/P_0_. Also, the isotherms show a large N_2_ uptake under low relative pressures, suggesting the abundant existence of micropores. The distinct hysteresis of the desorption curves at P/P_0_ = 0.4–1.0 also reveal the presence of mesopores and macropores. The pore size distribution is shown in Figure 2d. Obviously, FWR and FWRA have a wide pore size distribution of 2.0–100 nm. The porosity characteristics are summarized in Table 1. Compared with WR, the fungi treated FWR and FWRA shows a considerably enlarged specific surface area. The specific surface area of FWR and FWRA is 608.50 m^2^ g^−1^ and 1,165.68 m^2^ g^−1^ respectively, whereas that of WR is only 336.72 m^2^ g^−1^, indicating that the fungal pretreatment has an effect of increasing the specific surface area. Moreover, the fungi pretreatment in combination with NaCl activation further increases the surface area. 

### 3.3. Chemical Composition

**Figure 3:**
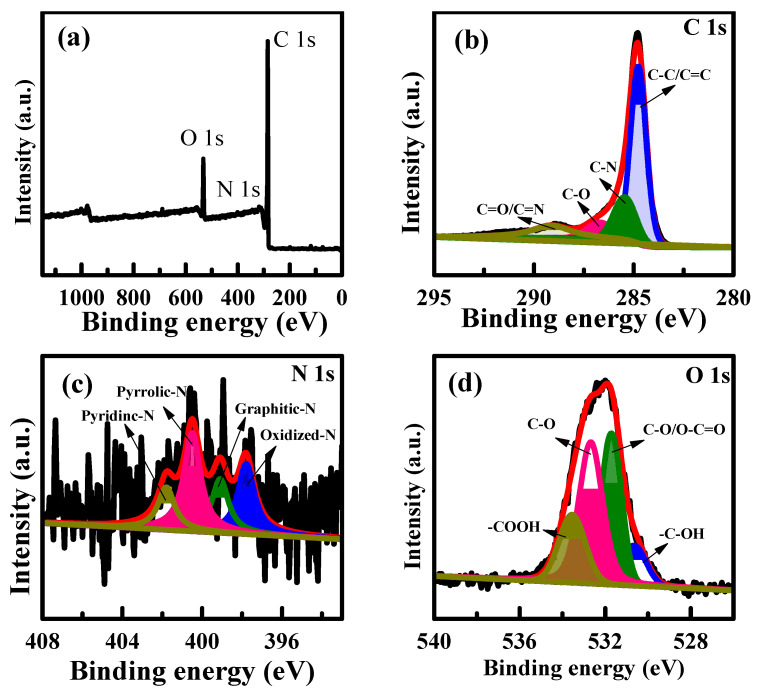
XPS analysis of FWRA sample: (**a**) Survey scan; (**b**) C1s; (**c**) N1s and (**d**) O1s.

The chemical compositions of the FWRA sample were examined by XPS. The survey spectra show the C1s (285 eV), O1s (533 eV) and N1s (400 eV) signals (Figure 3a). In the C1s spectrum with deconvolution(Figure 3b), the peaks at 284.7, 285.4, 286.7 and 289.1 eV are ascribable to C-C/C=C, C-N, C-O and C=O/C=N species, respectively [36]. The N1s profile can be resolved into four peaks (Figure 3c), the peaks at around 397.8, 399.1, 400.5 and 401.8 eV are assigned to pyridinic N, pyrrolic N, graphitic-N and oxidized-N, respectively. The O 1s spectrum is presented in Figure 3d and shows four distinct peaks at 530.6, 531.7, 532.6 and 533.5 eV, assignable to C-OH, C-O, O-C=O and COOH groups, respectively [37]. These results suggest the existence of abundant N-, and O- functional groups in FWRA.The functional groups provide a large number of defects which leads to the formation of active sites.

### 3.4. Electrochemical Performance

**Figure 4:**
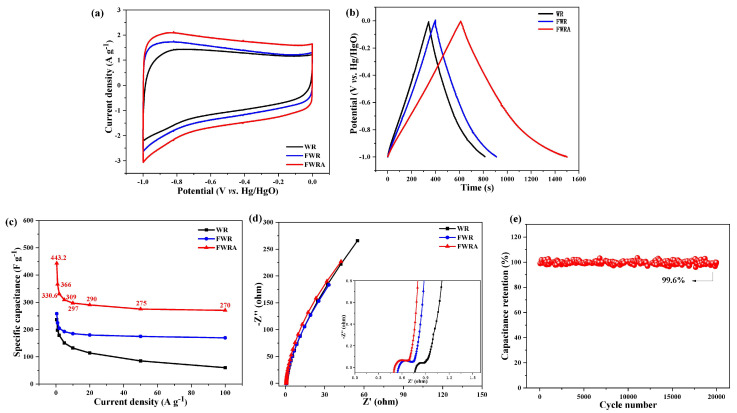
Electrochemical performances of the WR, FWR and FWRA electrodes tested in 6 M KOH electrolyte: (**a**) CV curves at a scan rate of 10 mV s^−1^; (**b**) GCD curves at a current density of 1.0 A g^−1^; (**c**) specific capacitance versus current density; (**d**) Nyquist plots of the electrodes fabricated based on WR, FWR and FWRA and the inset is the magnified image of high-frequency region; (**e**) Cycling stability at a current density of 5 A g^−1^ for the FWRA sample.

The energy storage performances of WR, FWR and FWRA were examined in 6 M KOH electrolyte employing a three-electrode system. The CV curves of all samples exhibited a quasi-rectangular shape in the potential range of −1.0 to 0 V, showing that the area represents the capacitance of the carbon materials (Figure 4a). 

At the same time, the GCD curves of all samples maintained a typical symmetrical shape at 1.0 A/g current densities (Figure 4b), indicating the materials have small resistance, good reversibility and high coulombic efficiencies. It is worth noting that the GCD curve of FWRA is slightly deformed, which is indicative of the existence of pseudocapacitance besides double layer capacitance. This is due to the nitrogen- and oxygen-containing functional groups in FWRA [38,39]. As shown in Figure 4c, the capacitances of the electrodes calculated according to the discharge curves (0.5 A/g) were 236, 258 and 443.2 F/g for WR, FWR and FWRA, respectively. 

The capacitive behaviors of all samples were further studied by EIS. As shown in Figure 4d, the steep linear curves in the low frequency region indicate almost ideal capacitive performance, which can promote the rapid transmission of electrons and diffusion of electrolytes. In the high frequency region, the semicircle represents the charge-transfer resistance, which ranged from 0.49 to 0.76 Ω, suggesting small ion diffusion resistance [40]. Furthermore, the GCD measurement was used to evaluate the cycle stability of FWRA at 5.0 A/g. As shown in Figure 4e, the capacitance retention is excellent, close to 99.6% after 20,000 cycles.

### 3.5. Methyl Orange Adsorption Properties 

**Figure 5:**
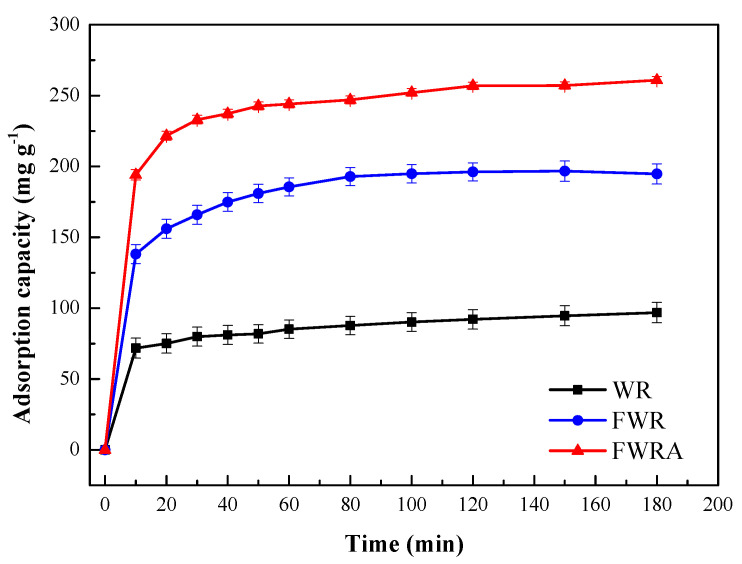
Adsorption performance of WR, FWR and FWRA. (MO of 50 mg/L, adsorbent of 12.5 mg (0.25 g/L), and 25 °C).

In addition to be promising as an electrode material for supercapacitors, FWRA is an efficient adsorbent. Methyl orange (MO) was used as a probe, and the results of UV absorption experiments for WR, FWR and FWRA are shown in Figure 5. It can be seen that FWRA (about 260.8 mg g^−1^) exhibited the best MO adsorption capacity compared to WR sample (less than 100 mg g^−1^). 

## 4. Discussion

Biomass-derived carbon-based materials are receiving extensive attention nowadays. The huge amount of traditional Chinese medicine residue is not only underutilized, but also an environmental issue. Therefore, we assessed whether the residues of traditional Chinese medicine could be used as carbon source for the generation of porous carbon versatile functionalities. Inspired by the degradation of biomass by white rot fungi in nature, we employed the biological pre-pore-forming method to facilitate the preparation of porous carbons that are in a high specific surface area. In this work, we deployed the special degradation capability of *P. vitreus* and obtained high performance FWRA by fungal pretreatment of wormwood rod residues via a simple carbonization and NaCl activation process.

Compared to the porous carbon prepared directly from raw biomass, the porous carbon that was prepared from the biomass pretreated by *P. vitreus* exhibits higher porosity and larger specific surface area. The phenomenon could be attributed to that the white-rot fungi can degrade plant-cell-wall components and loosen the internal structure of biomass. Therefore, during NaCl activation after the carbonization step, NaCl can have easy access to the internal surface of carbons for full activation [21,41,42]. The SEM results suggest that the FWR materials are much looser in structure than WR, whereas the FWRA material is the most significant in terms of porosity. The results imply that fungal pretreatment plays a key role in the forming of a porous structure. As indicated by the Raman results, FWRA is the lowest in graphitization degree, plausibly due to the high formation of pores. From the textual parameters of Table 1, it is clear that both the fungal pretreatment and NaCl activation steps are critical for the development of porosity in carbon.

The results of electrochemical performance reveal that the obtained FWRA has the nature of an electrochemical double-layer capacitor (EDLC). As an electrode material, FWRA displays maximum capacitance at a scan rate of 10 mV/s, showing an obvious redox peak on the CV curve. At a high current density of 100 A/g, FWRA exhibits high capacitance of 270 F/g (with 60.9% retention at 0.5 A/g). Since faradaic pseudocapacitance is caused by redox reaction between nitrogen-oxygen-containing functional groups and electrolyte [43,44]. The FWRA carbon with well-developed pore channels and large specific surface area is high in capacitance, even higher than a lot of reported carbon materials (Table 2). 

In addition to being a promising electrode material for supercapacitors, the FWRA carbon exhibited a high adsorption capacity towards methyl orange in aqueous solution. Among WR, FWR and FWRA, the adsorption capacity increased as a result of fugal pretreatment and NaCl activization. This is in agreement with the data in Table 1 that adsorption capacity increased with the increase of BET surface area and pore volume. It is obvious that the porous structure created by fungal pretreatment significantly improved the adsorption performance of the prepared carbons. Overall, the hyphae of white rot fungi infiltrates and biodegrades the internal structure of biomass substrate, enhancing the efficiency of the subsequent carbonization and chemical activation steps [7,21,28]. 

Generally speaking, the excellent performance of FWRA material can be accredited to the fungal pretreatment and NaCl activation steps. With high specific surface area, the porous carbon is versatile in practical applications. This study not only promotes the utilization of traditional Chinese medicine residues by turning them to valuable carbons, but also provides inspiration for performance optimization of porous carbons. Subsequently, the conditions of porous carbon prepared from fungi pretreated biomass can be further optimized to improve performance. Moreover, the universality of the fungal pretreatment for the fabrication of porous carbon was necessary to further investigation of, for example, the matching of fungi type and biomass nature. 

## 5. Conclusions

In summary, fungal pretreatment provides a powerful pathway to obtain versatile porous carbon materials from wormwood rod residues via a simple carbonization and activation process (FWRA strategy). The as-obtained FWRA carbon has high specific surface areas of 1165.7 m^2^ g^−1^ and abundant pores in its hierarchical structure. Due to wonderful properties, the FWRA carbon offered a high specific capacitance of 443.2 F g^−1^ at 0.5 A g^−1^ and outstanding ability of keeping a specific capacitance rate of 270 F g^−1^ at 100 A g^−1^ in 6 M KOH electrolyte. The symmetrical capacitor based on the carbon has superb cyclic stability, showing a low decay rate of specific capacitance less than 0.4% after 20,000 cycles at 5 A g^−1^ in 1 M Na_2_SO_4_ electrolyte. Moreover, in the removal of methyl orange (MO) from water, the FWRA-derived carbon displayed superior absorption performance (260.8 mg g^−1^), rendering it an ideal biochar adsorbent. Overall, the results of the present study demonstrate that the FWRA strategy has broad application prospects. It is envisioned that it can be utilized in the disposal of lignocellulosic materials for the generation of versatile porous carbons promising for energy storage and pollution remedy.

## Figures and Tables

**Table 1:** Pore parameters of samples.

Samples	S_BET_ (m^2^ g^−1^)	S_micro_ (m^2^ g^−1^)	V_total_ (cm^3^ g^−1^)	V_micro_ (cm^3^ g^−1^)
WR	336.7 ± 9.2	296.7 ± 7.2	0.28 ± 0.02	0.14 ± 0.01
FWR	608.5 ± 11.6	476.8 ± 7.9	0.56 ± 0.02	0.23 ± 0.02
FWRA	1165.7 ± 12.4	778.5 ± 8.2	1.02 ± 0.03	0.39 ± 0.02

**Table 2:** Comparison of electrochemical performance of porous carbon materials derived from different biomaterials.

Electrode Materials	Precursor	Electrolyte	Specific Capacitance (F g^−1^)	Cycling Stability	Ref.
Hollow tubular porous carbon	Straw cellulose waste	6 M KOH	312.57 F g^−1^ at 0.5 A g^−1^	92.93 % after 20,000 cycles	[35]
Porous carbon	Aniline-modified fungus	6 M KOH	218 F g^−1^ at 0.1 A g^−1^	100 % after 5000 cycles	[45]
Nitrogen-Doped Hierarchical Meso/Microporous Carbon	Bamboo Fungus	2 M KOH	228 F g^−1^ at 0.5 A g^−1^	100 % after 10,000 cycles	[46]
Hierarchically porous carbon nanosheets	Hierarchically porous carbon nanosheets	6 M KOH	283 F g^−1^ at 0.5 A g^−1^	95 % after 20,000 cycles	[47]
Hierarchical micro-meso-macro-porous carbon	Shaddock endotheliums	1 M H_2_SO_4_	550 F g^−1^ at 0.2 A g^−1^	93.7 % after 10,000 cycles	[48]
Porous carbon	Rape pollen	Rape pollen	390 F g^−1^ at 0.5 A g^−1^	92.9 % after 10,000 cycles	[49]
Porous N-Doped Carbon-Zinc Manganese Oxide Nanocomposite	Fungus Bran	3 M KOH	537 F g^−1^ at 1 A g^−1^	-	[15]
Biomass-derived carbons	Flammulina velutipes	1 M Na_2_SO_4_	167 F g^−1^ at 0.5 A g^−1^	98.7 % after 10,000 cycles	[50]
Porous biomass carbon materials	Cherry stones	6 M KOH	370.5 F g^−1^ at 0.5 A g^−1^	99.1 % after 5000 cycles	[51]
Oxygen-rich porous carbons	Alfalfa flowers	6 M KOH	350.1 F g^−1^ at 0.5 A g^−1^	-	[52]
Porous carbon	Sorghum	2 M KOH	257.2 F g^−1^ at 0.1 A g^−1^	-	[53]
FWRA	wormwood rod	6 M KOH	443.2 F g^−1^ at 0.5 A g^−1^	99.6 % after 20,000 cycles	This work

## Data Availability

Not applicable.
